# Dimensional specificity of foreign language enjoyment in mediating AI-assisted informal digital learning and L2 willingness to communicate: evidence from Chinese University learners

**DOI:** 10.3389/fpsyg.2026.1817760

**Published:** 2026-05-04

**Authors:** Sisi Guo, Chongkai Wang

**Affiliations:** School of Languages and Culture, Hebei GEO University, Shijiazhuang, China

**Keywords:** AI-assisted language learning, foreign language enjoyment, informal digital learning, positive psychology, willingness to communicate

## Abstract

**Introduction:**

The proliferation of generative artificial intelligence (AI) has created unprecedented opportunities for informal second language (L2) learning, yet the psychological mechanisms through which AI-mediated learning translates into communicative engagement remain poorly understood. This study examines how AI-mediated informal digital learning of English (AI-IDLE) shapes learners' willingness to communicate (WTC) in an L2 through the dimensional specificity of foreign language enjoyment (FLE).

**Methods:**

A large-scale investigation was conducted with 1,362 non-English-major university students in China. Using structural equation modeling with confirmatory factor analysis, parallel mediation models were estimated in which receptive and productive AI-IDLE predicted in-class and out-of-class L2 WTC through three FLE dimensions simultaneously: teacher appreciation, personal enjoyment, and social enjoyment.

**Results:**

FLE partially mediated the AI-IDLE-WTC link, but the three FLE dimensions operated through context-dependent pathways. Personal enjoyment emerged as a robust cross-contextual mediator in both classroom and extracurricular settings, whereas teacher appreciation mediated the relationship exclusively in structured classroom environments and showed no significant indirect effect in out-of-class contexts. Social enjoyment contributed to mediation across both settings, but with markedly attenuated strength outside the classroom. Productive AI-IDLE exhibited stronger associations with all FLE dimensions than receptive AI-IDLE.

**Discussion:**

These findings advance a dimensional specificity framework for understanding how positive emotions differentially channel technology-enhanced learning into communicative behavior. The results carry implications for the design of AI learning tools that deliberately target distinct affective pathways and underscore the importance of context-sensitive approaches to fostering L2 willingness to communicate.

## Introduction

Generative AI technologies, exemplified by large language models such as ChatGPT, have fundamentally altered the landscape of L2 learning (Cai, 2023; Mehdi, 2023; Rudolph et al., 2023; Yan, 2023). These systems provide real-time grammatical feedback, simulate authentic conversational contexts, and deliver personalized scaffolding that adapts to individual proficiency levels, thereby extending L2 practice well beyond the spatial and temporal constraints of traditional classrooms (Cong-Lem et al., 2025; Kohnke et al., 2023). The resulting mode of learning, termed AI-IDLE, enables learners to engage autonomously with the target language through receptive activities (e.g., AI-curated listening and reading) and productive activities (e.g., AI-assisted dialogue and writing), fulfilling the criteria of learner-initiated, unstructured, and digitally situated practice outlined in Benson's four-dimensional framework of out-of-class language learning (Benson, 2011; Lee and Hsieh, 2019). Unlike conventional IDLE, which involves learner-initiated engagement with fixed digital content, AI-IDLE features generative systems that dynamically adapt to learner input, provide real-time feedback, and simulate responsive conversational exchange, affording a qualitatively different structure of engagement with direct implications for which affective pathways are activated. Despite rapid adoption, the psychological mechanisms by which AI-IDLE influences downstream communicative behavior remain insufficiently theorized.

A growing body of work situated within positive psychology (PP) in second language acquisition (SLA) has identified foreign language enjoyment (FLE) as a pivotal affective construct that energizes learning engagement and predicts communicative outcomes (Dewaele, 2022; Dewaele and MacIntyre, 2014; Li et al., 2018). Grounded in Fredrickson's broaden-and-build theory (Fredrickson, 2001, 2003), FLE is posited to expand learners' momentary thought–action repertoires and accumulate durable personal resources, including social bonds, linguistic confidence, and communicative resilience, that collectively facilitate WTC in the L2 (Balouchi and Samad, 2021; Lewis et al., 2016; MacIntyre et al., 2019). L2 WTC, defined as the readiness to enter into discourse at a particular moment with a specific interlocutor (MacIntyre et al., 1998), occupies a central position in MacIntyre et al.'s pyramid model as both a proximal antecedent of actual communication and a key non-linguistic outcome of the learning process (MacIntyre et al., 1998; Nematizadeh, 2021). Empirical evidence consistently shows that learners who report higher FLE also exhibit greater L2 WTC (Alrabai, 2024; Dewaele and Pavelescu, 2019; Zhang et al., 2024), yet the directionality of this relationship and its boundary conditions across different learning ecologies have received limited scrutiny.

Critically, FLE is not a monolithic construct. Botes et al.'s validated short-form scale decomposes FLE into three empirically separable dimensions: *teacher appreciation* (positive appraisals of instructor support), *personal enjoyment* (intrinsic pleasure derived from the learning process), and *social enjoyment* (satisfaction from peer interaction) (Botes et al., 2021). This tripartite structure carries important theoretical implications: if different FLE dimensions are differentially activated by AI-IDLE and differentially predictive of WTC across contexts, then composite FLE scores, the default in most prior work (Liu et al., 2025a,b), mask meaningful heterogeneity in the affective pathways linking technology use to communicative behavior. We term this the *dimensional specificity hypothesis*: the proposition that distinct FLE dimensions serve as context-dependent mediators, each channeling the effects of AI-IDLE onto L2 WTC through qualitatively different psychological mechanisms. Although teacher appreciation's absence in out-of-class contexts may seem intuitively predictable, the hypothesis is non-trivial in two respects: prior work using composite FLE scores has never empirically verified this differential pattern, and the hypothesis extends to the relative mediating strengths of personal and social enjoyment across contexts, predictions that are neither logically necessary nor previously established.

Three gaps in the existing literature motivate the present study. First, although several investigations have documented positive associations between informal digital learning and both FLE and L2 WTC (Lee, 2022; Lee et al., 2023a; Wu, 2024; Zhang et al., 2024), the majority employ composite FLE measures that preclude dimension-level inference. Recent work has further extended AI-IDLE research by examining learners' beliefs through Q methodology and by integrating L2 self-guides and achievement emotions into network-analytic frameworks, underscoring the complexity of affective pathways in AI-mediated learning (Wang et al., 2025, 2026). The few studies that do disaggregate FLE dimensions (Dewaele et al., 2018; Lee et al., 2023b) have not simultaneously modeled receptive and productive AI-IDLE as distinct predictor facets, leaving open the question of whether the mode of AI engagement (input-oriented vs. output-oriented) differentially activates specific affective pathways. Second, existing work overwhelmingly examines WTC in a single context, typically the classroom, without comparing in-class and out-of-class WTC within the same analytical framework (Denies et al., 2015; Wang et al., 2021). Given that MacIntyre et al.'s pyramid model emphasizes situational variability as a defining feature of WTC (MacIntyre et al., 1998), a dual-context design is essential for testing whether the mediating architecture of FLE dimensions remains invariant or shifts across structured and unstructured communicative settings. Third, most prior studies rely on regression-based mediation with manifest variables (Wu, 2024; Zhang et al., 2024), which neither accounts for measurement error nor provides global model-fit indices. Structural equation modeling (SEM) with latent variables offers a methodologically stronger platform for testing multi-mediator models while simultaneously evaluating the measurement integrity of each construct (Kline, 2023).

To provide a more comprehensive theoretical account, we integrate Fredrickson's broaden-and-build theory with self-determination theory (SDT) (Deci and Ryan, 1985; Ryan and Deci, 2000). SDT posits that the satisfaction of three basic psychological needs (autonomy, competence, and relatedness) constitutes the proximal mechanism through which contextual factors generate positive affect (Ryan and Deci, 2000). AI-IDLE features map onto these needs with notable specificity: personalized content selection and self-paced practice fulfill autonomy needs, activating personal enjoyment; real-time corrective feedback and adaptive difficulty scaffolding enhance perceived competence, potentially amplifying all three FLE dimensions; and interactive features such as AI-matched discussion communities satisfy relatedness needs, nurturing social enjoyment (Lee et al., 2023b; Seddik, 2025). Teacher appreciation, by contrast, is hypothesized to be activated primarily in contexts where AI augments visible instructor involvement (e.g., AI-assisted personalized assignments) rather than in fully autonomous out-of-class settings (Rezai et al., 2024). It should be noted that this theoretical mapping is necessarily speculative, as basic psychological needs were not directly measured in the present study. The SDT-based reasoning is therefore offered as an interpretive scaffold rather than a tested causal account, and future research explicitly measuring autonomy, competence, and relatedness satisfaction would be required to verify the proposed mechanisms.

The present study tests the dimensional specificity hypothesis with a sample of 1,362 non-English-major university students across multiple institutions in China. We employ structural equation modeling to estimate parallel mediation models in which receptive and productive AI-IDLE predict in-class and out-of-class L2 WTC through the three FLE dimensions simultaneously. Six sets of hypotheses are tested: teacher appreciation (H1/H4), personal enjoyment (H2/H5), and social enjoyment (H3/H6) each mediate the AI-IDLE–WTC link for in-class and out-of-class contexts, respectively. By disaggregating both the predictor (receptive vs. productive AI-IDLE) and the mediator (three FLE dimensions) while contrasting two communicative contexts, this design offers a fine-grained map of the affective architecture connecting AI-assisted learning to communicative willingness, a level of analytical resolution that, to our knowledge, has not been achieved in prior work. The study thereby makes three principal contributions: it introduces and tests the dimensional specificity framework for FLE; it extends the theoretical integration of broaden-and-build theory and SDT to AI-mediated informal learning; and it advances the situational variability thesis in WTC theory through a dual-context analytical design.

## Methods

### Ethical approval

This study was reviewed and approved by the Academic Ethics Committee of School of Languages and Culture, Hebei GEO University. All procedures complied with the Declaration of Helsinki. Written informed consent was obtained from every participant prior to data collection.

### Participants

A total of 1,562 first- and second-year non-English-major undergraduates were recruited from three universities in Hebei Province, Mainland China, during the spring semester of 2025. The three institutions were selected to represent different university tiers within the Hebei Province system, including one comprehensive research university, one applied science and technology university, and one teaching-oriented university, in order to enhance the disciplinary and institutional diversity of the sample. Recruitment followed a stratified cluster sampling strategy in which intact classes were selected proportionally across engineering, science, business, liberal arts, and arts programmes to ensure disciplinary diversity.

For the study targeted AI-mediated informal learning, respondents who reported never having used generative AI tools for English learning were excluded (*n* = 200), yielding a final analytic sample of 1,362 participants. The excluded participants were by definition non-users whose AI-IDLE scores would have been effectively zero, introducing a structural floor spike rather than plausible variance in the predictor. The exclusion therefore reflects the theoretical scope of the study, which concerns the affective consequences of AI-IDLE among active users. Descriptive statistics for both the full sample and the excluded non-users are reported in Supplementary Table S1.

Participants reported using a variety of generative AI tools for English learning, with the most frequently mentioned including Deepseek, Doubao (a Chinese AI assistant), Ernie Bot (Wenxin Yiyan), and AI-powered language learning apps such as Duolingo and Liulishuo. These tools were used for both receptive activities (e.g., AI-generated content recommendations) and productive activities (e.g., conversational practice with AI chatbots, AI-assisted writing). No restrictions were placed on specific platforms, as the study aimed to capture the breadth of learners' AI-mediated informal learning experiences.

Table 1 summarizes the demographic characteristics of the sample. The age range was 17 to 30 years, with the majority aged 19 (40.5%) or 20 (30.0%). Females accounted for 57.6% of respondents. Most participants were freshmen (78.6%), self-assessed their English proficiency as beginner level (76.4%), and used AI tools one to two times per week (40.1%) or one to two times per month (39.6%).

**Table 1:** Demographic characteristics of the participants (*N* = 1,362).

Variable	Category	*n*	%
Age	17	5	0.4
	18	318	23.3
	19	551	40.5
	20	408	30.0
	21	60	4.4
	22	17	1.2
	≥23	3	0.2
Gender	Male	578	42.4
	Female	784	57.6
Academic year	Freshman	1,070	78.6
	Sophomore	288	21.1
	Junior	4	0.3
Major	Engineering	560	41.1
	Science	184	13.5
	Business	214	15.7
	Liberal arts	138	10.1
	Arts	218	16.0
	Other	48	3.5
Self-assessed English proficiency	Beginner	1,040	76.4
	Intermediate	306	22.5
	Upper-intermediate	6	0.4
	Advanced	10	0.7
Frequency of AI usage	Daily	98	7.2
	3 to 4 times per week	178	13.1
	1 to 2 times per week	546	40.1
	1 to 2 times per month	540	39.6

### Instruments

An online questionnaire comprising four sections was administered. All attitudinal items employed a five-point Likert scale (1 = strongly disagree; 5 = strongly agree). To safeguard data quality, one attention-check item was embedded in the AI-IDLE section, and questionnaires with incorrect responses were removed before analysis.

*AI-IDLE*. Eight items adapted from the IDEAL questionnaire (Zhang and Liu, 2024) measured the frequency of receptive and productive English learning activities with AI assistance. Receptive AI-IDLE (four items) captured input-oriented activities such as using AI to discover English songs, reading materials, videos, and podcasts. Productive AI-IDLE (four items) assessed output-oriented activities including conversational practice with AI chatbots, AI-assisted writing, pronunciation correction, and discussion prompt generation. Item wording was modified to foreground the AI-mediated nature of each activity (e.g., the original item “I listen to English songs” was revised to “I ask AI to recommend English songs matching my preferences to improve my English listening skills”). Cronbach's α was 0.90 for the receptive subscale and 0.91 for the productive subscale. Prior to the main data collection, the adapted items were reviewed by two bilingual researchers in applied linguistics who confirmed that the revised wording clearly communicated AI-mediated activities and was unlikely to be conflated with general digital learning behaviors.

*FLE*. Nine items from the short-form FLE scale (Botes et al., 2021) were adopted and adapted to measure three subdimensions: teacher appreciation (three items; e.g., “The teacher is encouraging”), personal enjoyment (three items; e.g., “I enjoy learning English”), and social enjoyment (three items; e.g., “We share common topics in English class”). Minor wording adjustments were made for cultural clarity; for instance, the social enjoyment item “We have common legends, such as running jokes” was revised to “We share common topics in English class” because the concept of “legends” was deemed potentially ambiguous for mainland Chinese students, while teacher appreciation and personal enjoyment items were retained without modification as they were judged to be culturally appropriate. Cronbach's α values were 0.89, 0.87, and 0.86 for teacher appreciation, personal enjoyment, and social enjoyment, respectively.

*L2 WTC*. Six items adapted from (Lee et al. 2023b) measured WTC across two contexts. In-class L2 WTC (three items) assessed willingness to speak English during class discussions, volunteer to answer questions, and deliver presentations. Out-of-class L2 WTC (three items) assessed willingness to communicate in English in everyday settings such as cafés, shopping malls, and social events. Item scenarios were adjusted to better reflect the communicative contexts typically available to mainland Chinese university students. Cronbach's α was 0.83 for in-class WTC and 0.84 for out-of-class WTC.

### Procedure

Following ethical approval, the research team distributed an online questionnaire via the Wenjuanxing platform (www.wjx.cn) to participants across the three participating institutions. Data collection spanned 4 weeks. Participants were briefed on the study's objectives, procedures, and confidentiality provisions and provided informed consent before proceeding. The median completion time was approximately 12 min. Responses were screened for straight-lining, excessively short completion times (below 3 min), and incorrect attention-check answers, resulting in the exclusion of 200 cases.

### Analytic strategy

Analyses proceeded in four stages. First, preliminary screening examined data normality, missing values, and common method bias. Because all variables were assessed via a single self-report questionnaire administered at one time point, we tested for common method variance (CMV) using both Harman's single-factor test and a confirmatory factor analysis (CFA) approach in which a latent common method factor was added to the measurement model (Podsakoff et al., 2003). Second, the measurement model was evaluated through CFA to establish construct validity. Model fit was assessed using the chi-square statistic (χ^2^), comparative fit index (CFI), Tucker-Lewis's index (TLI), root mean square error of approximation (RMSEA), and standardized root mean square residual (SRMR). Following conventional benchmarks (Kline, 2023), acceptable fit was defined as CFI and TLI ≥0.95, RMSEA ≤ 0.06, and SRMR ≤ 0.08. Convergent validity was assessed through composite reliability (CR ≥0.70) and average variance extracted (AVE ≥0.50); discriminant validity was confirmed when the square root of each construct's AVE exceeded its correlations with all other constructs (Fornell and Larcker, 1981). Third, alternative measurement models (five-factor and one-factor) were compared against the hypothesized seven-factor model to verify the distinctiveness of the constructs. Fourth, structural equation modeling (SEM) with maximum likelihood estimation was used to test the hypothesized mediation pathways. Separate structural models were estimated for in-class and out-of-class L2 WTC as the outcome variable, with receptive and productive AI-IDLE as exogenous predictors and the three FLE dimensions as parallel mediators. Indirect effects were evaluated using bias-corrected bootstrapping with 5,000 resamples, and a 95% confidence interval excluding zero was taken as evidence of significant mediation (Preacher and Hayes, 2008). Following prior research (Lee, 2022), age, gender, and academic year were included as covariates in all structural models to control for potential demographic confounds. All CFA and SEM analyses were conducted in Mplus 8.3 (Muthén and Muthén, 2017) and descriptive statistics were computed in SPSS 28.0. It should be noted that the paths from AI-IDLE to the three FLE dimensions are numerically identical across the two structural models, as both are estimated from the same dataset; the theoretical interest lies in comparing the FLE-to-WTC indirect effects across in-class and out-of-class outcome contexts, not in treating the predictor-to-mediator paths as independently replicated findings.

### Preliminary analyses

Inspection of univariate distributions confirmed that skewness values ranged from −0.55 to 0.86 and kurtosis values from −0.90 to −0.06, all within acceptable thresholds for maximum likelihood estimation. No variable had more than 1.2% missing data, and Little's MCAR test was non-significant (χ^2^ = 218.43, *df* = 197, *p* =0.14), supporting the assumption that data were missing completely at random; missing values were handled via full information maximum likelihood. Harman's single-factor test indicated that the first unrotated factor accounted for 28.6% of total variance, well below the 40% threshold. In the CFA-based CMV test, adding a common method factor did not substantially improve model fit (ΔCFI = 0.008, ΔRMSEA = 0.003), and all factor loadings remained significant and substantively unchanged, suggesting that common method bias was unlikely to pose a serious threat to the validity of the findings.

### Measurement model

The hypothesized seven-factor CFA model (receptive AI-IDLE, productive AI-IDLE, teacher appreciation, personal enjoyment, social enjoyment, in-class WTC, and out-of-class WTC) demonstrated good fit to the data: χ^2^(209) = 524.37, χ^2^/*df* = 2.51, CFI = 0.962, TLI = 0.955, RMSEA = 0.041 (90% CI [0.036, 0.046]), SRMR = 0.033. All standardized factor loadings were significant and ranged from 0.76 to 0.88 (Table 2), exceeding the recommended minimum of 0.60. Two alternative models were tested for comparison: a five-factor model that merged the three FLE dimensions into a single factor and combined the two WTC contexts into a single factor, and a one-factor model that loaded all items onto a single latent variable. Both alternative models exhibited substantially poorer fit (Table 3), confirming that the seven constructs are empirically distinct.

**Table 2:** Standardized factor loadings from confirmatory factor analysis.

Construct	Item	Description	λ
AI-IDLE (receptive)	AI-R1	I ask AI to recommend English songs matching my preferences	0.78
	AI-R2	I use AI to find English reading materials suited to my level	0.82
	AI-R3	I watch English videos recommended by AI algorithms	0.76
	AI-R4	I use AI to discover English podcasts on topics I enjoy	0.80
AI-IDLE (productive)	AI-P1	I practice English conversation with AI chatbots	0.84
	AI-P2	I use AI to help me write English essays or emails	0.81
	AI-P3	I ask AI to correct my English pronunciation	0.79
	AI-P4	I use AI to generate English discussion prompts for practice	0.83
FLE (teacher appreciation)	TA1	The teacher is encouraging	0.85
	TA2	The teacher is supportive	0.88
	TA3	The teacher is friendly	0.82
FLE (personal enjoyment)	PE1	I enjoy learning English	0.86
	PE2	English classes are a positive experience for me	0.83
	PE3	I have learnt interesting things in English class	0.80
FLE (social enjoyment)	SE1	We share common topics in English class	0.79
	SE2	We form a supportive group in English class	0.82
	SE3	There is a good atmosphere in English class	0.84
L2 WTC (in-class)	WI1	I am willing to speak English in class discussions	0.81
	WI2	I volunteer to answer English questions in class	0.78
	WI3	I am willing to do English presentations in class	0.76
L2 WTC (out-of-class)	WO1	I communicate in English when meeting foreign friends in a café	0.80
	WO2	I use English in shopping malls when the opportunity arises	0.77
	WO3	I speak English with foreign acquaintances at social events	0.82

All loadings significant at *p* < 0.001.

λ, standardized factor loading; AI-R, receptive AI-IDLE; AI-P, productive AI-IDLE; TA, teacher appreciation; PE, personal enjoyment; SE, social enjoyment; WI, in-class WTC; WO, out-of-class WTC.

**Table 3:** Model fit indices for measurement and structural models.

Model	χ^2^	df	χ^2^/df	CFI	TLI	RMSEA	SRMR
Measurement model (7-factor CFA)	524.37	209	2.51	0.962	0.955	0.041	0.033
Alternative model (5-factor)	1,287.64	214	6.02	0.891	0.876	0.078	0.061
Alternative model (1-factor)	4,532.18	219	20.70	0.584	0.547	0.142	0.109
Structural model (in-class WTC)	312.46	125	2.50	0.965	0.958	0.040	0.032
Structural model (out-of-class WTC)	298.73	125	2.39	0.968	0.961	0.038	0.031

χ^2^, chi-square; df, degrees of freedom; CFI, comparative fit index; TLI, Tucker-Lewis's index; RMSEA, root mean square error of approximation; SRMR, standardized root mean square residual.

Table 4 presents the reliability and validity indices. Composite reliability values ranged from 0.83 to 0.91, and AVE values ranged from 0.62 to 0.73, confirming adequate convergent validity across all constructs. Discriminant validity was supported by the Fornell-Larcker criterion: the square root of each construct's AVE (diagonal entries in Table 5) exceeded all corresponding inter-construct correlations. These results collectively indicate that the measurement model possesses satisfactory psychometric properties for structural analysis.

**Table 4:** Reliability and convergent validity of the measurement model.

Construct	Items	α	CR	AVE	√AVE
AI-IDLE (R)	4	0.90	0.90	0.70	0.84
AI-IDLE (P)	4	0.91	0.91	0.72	0.85
FLE-TA	3	0.89	0.89	0.73	0.85
FLE-PE	3	0.87	0.87	0.69	0.83
FLE-SE	3	0.86	0.86	0.67	0.82
L2 WTC-IN	3	0.83	0.83	0.62	0.79
L2 WTC-OUT	3	0.84	0.84	0.64	0.80

α, Cronbach's alpha; CR, composite reliability; AVE, average variance extracted; √AVE, square root of AVE for discriminant validity comparison.

**Table 5:** Descriptive statistics and inter-construct correlations.

Variable	M	SD	1	2	3	4	5	6
1. AI-R	3.28	0.91	**0.84**					
2. AI-P	3.47	0.96	0.51	**0.85**				
3. FLE-TA	4.24	0.72	0.34	0.39	**0.85**			
4. FLE-PE	3.49	0.94	0.46	0.52	0.41	**0.83**		
5. FLE-SE	3.98	0.82	0.38	0.44	0.47	0.53	**0.82**	
6. WTC-IN	3.51	0.97	0.29	0.39	0.35	0.48	0.42	**0.79**
7. WTC-OUT	2.80	1.05	0.35	0.40	0.12	0.43	0.30	0.51

All correlations significant at *p* < 0.001.

Bold diagonal values are the square root of AVE for each construct.

AI-R, receptive AI-IDLE; AI-P, productive AI-IDLE; FLE-TA, teacher appreciation; FLE-PE, personal enjoyment; FLE-SE, social enjoyment; WTC-IN, in-class WTC; WTC-OUT, out-of-class WTC.

Descriptive results (Table 5) showed that participants reported higher levels of productive AI-IDLE (*M* = 3.47, *SD* = 0.96) than receptive AI-IDLE (*M* = 3.28, *SD* = 0.91). Among the FLE dimensions, teacher appreciation received the highest mean endorsement (*M* = 4.24), followed by social enjoyment (*M* = 3.98) and personal enjoyment (*M* = 3.49). In-class WTC (*M* = 3.51) was substantially higher than out-of-class WTC (*M* = 2.80), consistent with the well-documented pattern of lower communicative willingness in unstructured settings among East Asian EFL learners (Denies et al., 2015; Wang et al., 2021).

### Structural model evaluation

Two separate structural models were estimated to test the hypothesized mediation pathways: one with in-class L2 WTC as the outcome and one with out-of-class L2 WTC as the outcome. In each model, receptive and productive AI-IDLE served as exogenous predictors, and the three FLE dimensions (teacher appreciation, personal enjoyment, and social enjoyment) were specified as parallel mediators. Age, gender, and academic year were included as covariates. Both models exhibited a good fit to the data. The in-class model yielded χ^2^(125) = 312.46, χ^2^/*df* = 2.50, CFI = 0.965, TLI = 0.958, RMSEA = 0.040, and SRMR = 0.032. The out-of-class model yielded χ^2^(125) = 298.73, χ^2^/*df* = 2.39, CFI = 0.968, TLI = 0.961, RMSEA = 0.038, and SRMR = 0.031 (see Table 3 in Methods). The structural results are detailed in Tables 6 and 7, and the proportional decomposition of effects is presented in Table 8. A summary of hypothesis testing outcomes is provided in Table 9.

**Table 6:** Standardized path coefficients and indirect effects for the in-class L2 WTC structural model.

Path	β	SE	*z*	*p*	95% CI
Direct paths					
AI-R → FLE-TA	0.31	0.04	7.75	< 0.001	
AI-R → FLE-PE	0.42	0.04	10.50	< 0.001	
AI-R → FLE-SE	0.35	0.04	8.75	< 0.001	
AI-P → FLE-TA	0.36	0.04	9.00	< 0.001	
AI-P → FLE-PE	0.48	0.04	12.00	< 0.001	
AI-P → FLE-SE	0.40	0.04	10.00	< 0.001	
FLE-TA → WTC-IN	0.22	0.03	7.33	< 0.001	
FLE-PE → WTC-IN	0.35	0.03	11.67	< 0.001	
FLE-SE → WTC-IN	0.28	0.03	9.33	< 0.001	
AI-R → WTC-IN (direct)	0.08	0.03	2.67	0.008	
AI-P → WTC-IN (direct)	0.12	0.03	4.00	< 0.001	
Indirect paths					
AI-R → FLE-TA → WTC-IN	0.068	0.013			[0.044, 0.095]
AI-R → FLE-PE → WTC-IN	0.147	0.019			[0.112, 0.186]
AI-R → FLE-SE → WTC-IN	0.098	0.015			[0.070, 0.129]
AI-P → FLE-TA → WTC-IN	0.079	0.013			[0.055, 0.106]
AI-P → FLE-PE → WTC-IN	0.168	0.020			[0.131, 0.209]
AI-P → FLE-SE → WTC-IN	0.112	0.016			[0.082, 0.145]
Total indirect effects					
AI-R → FLE (total) → WTC-IN	0.313	0.028			[0.260, 0.370]
AI-P → FLE (total) → WTC-IN	0.359	0.029			[0.304, 0.418]

Standardized coefficients reported.

SE, standard error; 95% CI, bias-corrected bootstrap confidence interval (5,000 resamples); AI-R, receptive AI-IDLE; AI-P, productive AI-IDLE; FLE-TA, teacher appreciation; FLE-PE, personal enjoyment; FLE-SE, social enjoyment; WTC-IN, in-class WTC.

**Table 7:** Standardized path coefficients and indirect effects for the out-of-class L2 WTC structural model.

Path	β	SE	*z*	*p*	95% CI
Direct paths					
AI-R → FLE-TA	0.31	0.04	7.75	< 0.001	
AI-R → FLE-PE	0.42	0.04	10.50	< 0.001	
AI-R → FLE-SE	0.35	0.04	8.75	< 0.001	
AI-P → FLE-TA	0.36	0.04	9.00	< 0.001	
AI-P → FLE-PE	0.48	0.04	12.00	< 0.001	
AI-P → FLE-SE	0.40	0.04	10.00	< 0.001	
FLE-TA → WTC-OUT	0.02	0.04	0.50	0.617	
FLE-PE → WTC-OUT	0.31	0.04	7.75	< 0.001	
FLE-SE → WTC-OUT	0.14	0.04	3.50	< 0.001	
AI-R → WTC-OUT (direct)	0.18	0.04	4.50	< 0.001	
AI-P → WTC-OUT (direct)	0.21	0.04	5.25	< 0.001	
Indirect paths					
AI-R → FLE-TA → WTC-OUT	0.006	0.013			[−0.019, 0.031]
AI-R → FLE-PE → WTC-OUT	0.130	0.021			[0.091, 0.173]
AI-R → FLE-SE → WTC-OUT	0.049	0.014			[0.023, 0.078]
AI-P → FLE-TA → WTC-OUT	0.007	0.014			[−0.020, 0.035]
AI-P → FLE-PE → WTC-OUT	0.149	0.023			[0.106, 0.196]
AI-P → FLE-SE → WTC-OUT	0.056	0.016			[0.027, 0.089]
Total indirect effects					
AI-R → FLE (total) → WTC-OUT	0.185	0.030			[0.128, 0.246]
AI-P → FLE (total) → WTC-OUT	0.212	0.032			[0.152, 0.277]

Standardized coefficients reported.

SE, standard error. 95% CI, bias-corrected bootstrap confidence interval (5,000 resamples). CI intervals including zero are considered non-significant. AI-R, receptive AI-IDLE; AI-P, productive AI-IDLE; FLE-TA, teacher appreciation; FLE-PE, personal enjoyment; FLE-SE, social enjoyment; WTC-OUT, out-of-class WTC.

**Table 8:** Proportional decomposition of direct and indirect effects across contexts.

Path	β (% of total)	Lower	Upper
In-class WTC			
AI-R → FLE-TA → WTC-IN	0.068 (17.4%)	0.044	0.095
AI-R → FLE-PE → WTC-IN	0.147 (37.6%)	0.112	0.186
AI-R → FLE-SE → WTC-IN	0.098 (25.1%)	0.070	0.129
AI-R → direct	0.080 (20.5%)		
AI-P → FLE-TA → WTC-IN	0.079 (16.5%)	0.055	0.106
AI-P → FLE-PE → WTC-IN	0.168 (35.1%)	0.131	0.209
AI-P → FLE-SE → WTC-IN	0.112 (23.4%)	0.082	0.145
AI-P → direct	0.120 (25.1%)		
Out-of-class WTC			
AI-R → FLE-TA → WTC-OUT	0.006 (1.6%) n.s.	−0.019	0.031
AI-R → FLE-PE → WTC-OUT	0.130 (35.6%)	0.091	0.173
AI-R → FLE-SE → WTC-OUT	0.049 (13.4%)	0.023	0.078
AI-R → direct	0.180 (49.3%)		
AI-P → FLE-TA → WTC-OUT	0.007 (1.7%) n.s.	−0.020	0.035
AI-P → FLE-PE → WTC-OUT	0.149 (35.6%)	0.106	0.196
AI-P → FLE-SE → WTC-OUT	0.056 (13.4%)	0.027	0.089
AI-P → direct	0.210 (50.2%)		

n.s., non-significant (95% bootstrap CI includes zero).

Percentages indicate the proportion of the total effect of AI-IDLE on L2 WTC accounted for by each pathway.

**Table 9:** Summary of hypothesis testing outcomes.

Hypothesis	Path	Indirect effect	Outcome
H1	FLE-TA mediates AI-IDLE → in-class WTC	AI-R: β = 0.068; AI-P: β = 0.079	Supported
H2	FLE-PE mediates AI-IDLE → in-class WTC	AI-R: β = 0.147; AI-P: β = 0.168	Supported
H3	FLE-SE mediates AI-IDLE → in-class WTC	AI-R: β = 0.098; AI-P: β = 0.112	Supported
H4	FLE-TA mediates AI-IDLE → out-of-class WTC	AI-R: β = 0.006; AI-P: β = 0.007 (n.s.)	**Not supported**
H5	FLE-PE mediates AI-IDLE → out-of-class WTC	AI-R: β = 0.130; AI-P: β = 0.149	Supported
H6	FLE-SE mediates AI-IDLE → out-of-class WTC	AI-R: β = 0.049; AI-P: β = 0.056	Supported

β = standardized indirect effect from bootstrap analysis (5,000 resamples).

n.s. = 95% confidence interval includes zero.

AI-IDLE encompasses both receptive and productive facets; effects for each facet are reported separately.

### Mediation pathways for in-class L2 WTC

Regarding in-class L2 WTC, both receptive and productive AI-IDLE exerted significant positive effects on all three FLE dimensions (Table 6). Productive AI-IDLE showed stronger associations with each FLE dimension than receptive AI-IDLE, with the largest path observed between productive AI-IDLE and personal enjoyment (β = 0.48, *p* < 0.001). All three FLE dimensions, in turn, significantly predicted in-class WTC: personal enjoyment exhibited the strongest effect (β = 0.35, *p* < 0.001), followed by social enjoyment (β = 0.28, *p* < 0.001) and teacher appreciation (β = 0.22, *p* < 0.001).

Bias-corrected bootstrap analyses confirmed that all six indirect effects through the three FLE dimensions were statistically significant, with 95% confidence intervals excluding zero. For the receptive AI-IDLE pathways, personal enjoyment carried the largest indirect effect (β = 0.147, 95% CI [0.112, 0.186]), accounting for 37.6% of the total effect. Social enjoyment mediated a further 25.1% (β = 0.098, 95% CI [0.070, 0.129]), and teacher appreciation mediated 17.4% (β = 0.068, 95% CI [0.044, 0.095]). The direct effect of receptive AI-IDLE on in-class WTC, although reduced in magnitude, remained significant (β = 0.08, *p* = 0.008), indicating partial mediation. The pattern for productive AI-IDLE was structurally parallel: personal enjoyment accounted for the largest share of the indirect effect (β = 0.168, 35.1%), followed by social enjoyment (β = 0.112, 23.4%) and teacher appreciation (β = 0.079, 16.5%), with a significant residual direct effect (β = 0.12, *p* < 0.001). These results provide support for H1, H2, and H3: all three FLE dimensions partially mediate the relationship between AI-IDLE and in-class L2 WTC, with personal enjoyment emerging as the pre-dominant mediating channel.

### Mediation pathways for out-of-class L2 WTC

For out-of-class L2 WTC, a markedly different mediating architecture emerged (Table 7). The paths from AI-IDLE to the three FLE dimensions were identical to those in the in-class model (as these are predictor-to-mediator paths shared across models). However, the FLE-to-WTC paths diverged substantially. Personal enjoyment retained a strong effect on out-of-class WTC (β = 0.31, *p* < 0.001), and social enjoyment showed a modest but significant effect (β = 0.14, *p* < 0.001). In contrast, teacher appreciation failed to predict out-of-class WTC (β = 0.02, *p* = 0.617), rendering its indirect pathway non-significant.

Bootstrap analyses confirmed this dimensional divergence. For receptive AI-IDLE, the indirect effect through personal enjoyment was significant and substantial (β = 0.130, 95% CI [0.091, 0.173], 35.6% of total effect), and the indirect effect through social enjoyment was smaller but significant (β = 0.049, 95% CI [0.023, 0.078], 13.4%). The indirect effect through teacher appreciation, however, was negligible and non-significant (β = 0.006, 95% CI [−0.019, 0.031]). The same pattern held for productive AI-IDLE: personal enjoyment (β = 0.149, 95% CI [0.106, 0.196]) and social enjoyment (β = 0.056, 95% CI [0.027, 0.089]) served as significant mediators, whereas teacher appreciation did not (β = 0.007, 95% CI [−0.020, 0.035]). Notably, the direct effects of both receptive AI-IDLE (β = 0.18, *p* < 0.001) and productive AI-IDLE (β = 0.21, *p* < 0.001) on out-of-class WTC were considerably larger than those observed in the in-class model, suggesting that AI-IDLE exerts a stronger unmediated influence on communicative willingness in informal settings. These results support H5 and H6 (personal enjoyment and social enjoyment mediate the AI-IDLE to out-of-class WTC relationship) but reject H4 (teacher appreciation does not mediate this relationship).

### Proportional decomposition of total effects

To facilitate cross-context comparison, Table 8 presents the proportional contribution of each pathway to the total effect of AI-IDLE on L2 WTC. Two patterns warrant attention. First, the total mediated proportion was markedly larger in the in-class context (approximately 75 to 80% of the total effect was channeled through FLE dimensions) than in the out-of-class context (approximately 49 to 51%), indicating that direct, unmediated pathways play a more prominent role when learners communicate outside structured classroom environments. Second, personal enjoyment consistently accounted for the single largest share of the indirect effect across both contexts and both AI-IDLE facets (35 to 38%), establishing it as the most robust cross-contextual mediator. Teacher appreciation contributed meaningfully in the classroom (16 to 17%) but was negligible outside the classroom, while social enjoyment made moderate contributions in both settings, though with reduced strength out of class (13 to 14% vs. 23 to 25% in class).

### Variance explained

The structural models explained substantial variance in the endogenous variables (Table 10). Among the FLE dimensions, personal enjoyment showed the highest explained variance (*R*^2^ = 0.39), followed by social enjoyment (*R*^2^ = 0.33) and teacher appreciation (*R*^2^ = 0.28), indicating that AI-IDLE more strongly predicts the experience of intrinsic learning pleasure and peer interaction satisfaction than perceptions of teacher support. The in-class WTC model accounted for 47% of the outcome variance, whereas the out-of-class WTC model accounted for 38%, consistent with the weaker total indirect effects observed in informal settings.

**Table 10:** Variance explained (*R*^2^) for endogenous variables.

Endogenous variable	*R* ^2^
FLE-TA	0.28
FLE-PE	0.39
FLE-SE	0.33
WTC-IN	0.47
WTC-OUT	0.38

Values represent the proportion of variance in each endogenous variable accounted for by the structural model including covariates.

### Summary of hypothesis tests

Table 9 provides a consolidated summary of the six hypothesis tests. Five of the six hypotheses were supported: the three FLE dimensions each mediated the AI-IDLE to in-class WTC link (H1, H2, H3), and personal enjoyment and social enjoyment mediated the AI-IDLE to out-of-class WTC link (H5, H6). The sole unsupported hypothesis was H4: teacher appreciation did not mediate the relationship between AI-IDLE and out-of-class WTC, as the indirect pathway through this dimension was statistically non-significant for both receptive and productive AI-IDLE. Taken together, the results confirm the dimensional specificity hypothesis proposed in the Introduction, demonstrating that the mediating architecture of FLE is not uniform but varies systematically as a function of the communicative context in which WTC is assessed.

**Figure 1:**
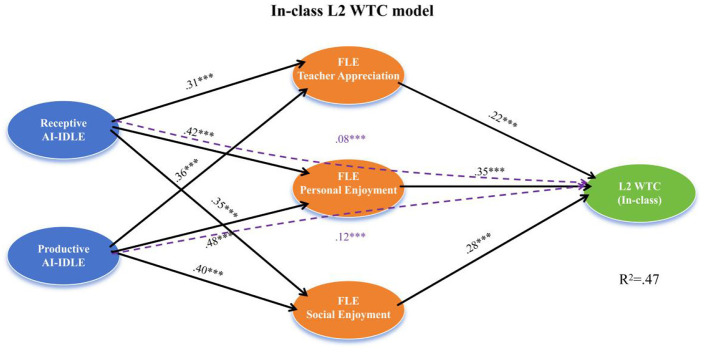
Structural model of FLE-mediated pathways from AI-IDLE to in-class L2 WTC. Standardized path coefficients are displayed. Solid lines indicate significant paths; dashed lines indicate non-significant paths. Covariates (age, gender, academic year) included but omitted for clarity. ***p < 0.001; n.s., non-significant.

As illustrated in Figure 1, both receptive and productive AI-IDLE exerted significant positive effects on all three FLE dimensions, which in turn predicted in-class L2 WTC. All three mediating pathways–through teacher appreciation, personal enjoyment, and social enjoyment–were statistically significant, supporting full-spectrum mediation in the classroom context. Personal enjoyment exhibited the largest indirect effect, followed by social enjoyment and teacher appreciation. Notably, significant direct effects from both AI-IDLE facets to in-class WTC remained after accounting for the mediators, indicating partial mediation.

**Figure 2:**
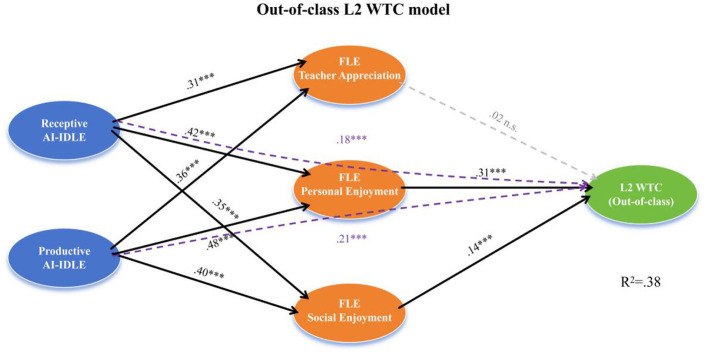
Structural model of FLE-mediated pathways from AI-IDLE to out-of-class L2 WTC. Standardized path coefficients are displayed. Solid lines indicate significant paths; dashed lines indicate non-significant paths. Covariates (age, gender, academic year) included but omitted for clarity. ***p < 0.001; n.s., non-significant.

Figure 2 depicts the structural model for out-of-class L2 WTC, revealing a contrasting mediating architecture. While the paths from AI-IDLE to the three FLE dimensions remained identical to those in the in-class model, the FLE-to-WTC paths diverged markedly. Personal enjoyment continued to serve as the strongest mediator, and social enjoyment retained a significant but attenuated mediating role. Crucially, teacher appreciation failed to predict out-of-class WTC, rendering its indirect effect non-significant—a finding that underscores the context-bound nature of this FLE dimension. Direct effects of AI-IDLE on out-of-class WTC remained significant, again confirming partial mediation.

## Discussion

This study set out to test the dimensional specificity hypothesis: that the three constituent dimensions of foreign language enjoyment (FLE) serve as context-dependent mediators in the pathway from AI-mediated informal digital learning of English (AI-IDLE) to second language willingness to communicate (L2 WTC). The structural equation modeling results, based on a sample of 1,362 Chinese university learners, provide robust support for this proposition. Five of six hypotheses were confirmed, and the single null finding (the absence of teacher appreciation as a mediator in out-of-class contexts) is itself theoretically informative. Below, we discuss the principal findings in relation to existing theory, highlight the theoretical and practical contributions, and acknowledge the limitations of the present work.

### Personal enjoyment as the cross-contextual core mediator

The most consistent finding was the primacy of personal enjoyment as a mediating channel. Across both in-class and out-of-class models, and for both receptive and productive AI-IDLE, personal enjoyment accounted for the largest share of the indirect effect (35 to 38% of total effects). This pattern is congruent with Fredrickson's broaden-and-build theory (Fredrickson, 2001, 2003), which predicts that intrinsic positive affect broadens cognitive and behavioral repertoires, and with self-determination theory's emphasis on intrinsic motivation as the most potent driver of sustained engagement (Deci and Ryan, 1985; Ryan and Deci, 2000). AI-IDLE environments afford learners considerable autonomy in selecting content, pacing, and difficulty level, conditions that are well documented to satisfy autonomy and competence needs and thereby generate the subjective experience of enjoyment (Lee et al., 2023b; Noels et al., 2000). The present results extend this reasoning by demonstrating that the enjoyment arising from such autonomous engagement does not merely correlate with WTC but actively transmits the effect of AI use onto communicative behavior regardless of whether communication occurs in a structured classroom or an informal setting.

The strength of this mediation pathway also illuminates why AI tools that prioritize personalisation and adaptive difficulty tend to outperform generic platforms in promoting communicative engagement (Zhang et al., 2024; Tai and Chen, 2023). When learners experience a sense of optimal challenge and perceive progress through AI-generated feedback, the resulting personal enjoyment functions as a psychological bridge between private learning activity and public communicative behavior. This mechanism is consistent with situated cognition theory: positive affect experienced during AI-mediated practice accumulates durable motivational resources that prime approach-oriented behavior in subsequent communicative situations.

### Teacher appreciation as a context-bound mediator

Teacher appreciation mediated the AI-IDLE to WTC link exclusively in the classroom (H1 supported) and showed no mediating function outside it (H4 rejected). This asymmetry is interpretable through the lens of situated cognition (Brown et al., 1989): perceptions of teacher support are formed and activated within the instructional context where teachers are physically and interactionally present. In out-of-class settings, the teacher is absent as an immediate social agent, and the affective residue of teacher appreciation appears insufficient to propel autonomous communicative behavior. This finding converges with earlier work showing that teacher-related FLE correlates more strongly with in-class than out-of-class outcomes (Dewaele and MacIntyre, 2014; Wang et al., 2021), and it refines this observation by specifying the mechanism: it is not that teacher appreciation is unrelated to AI-IDLE (AI-IDLE does predict teacher appreciation, β = 0.31 to 0.36), but rather that teacher appreciation fails to convert into communicative willingness when the communicative context lacks the teacher's scaffolding presence.

This result carries a nuance that warrants careful interpretation. It does not imply that teachers are irrelevant to out-of-class learning. Rather, it suggests that the affective pathway through which teacher-related emotions influence WTC is structurally dependent on the teacher's co-presence. When AI tools are used outside the classroom, any enhancement of teacher appreciation that occurs (for e.g., because a teacher recommended a particular AI tool) does not propagate forward to increase out-of-class communicative behavior. This stands in partial contrast to (Lai et al. 2016), who reported cross-context transfer of teacher influence among primary school learners in Hong Kong. The divergence may reflect developmental differences: university students, whose L2 motivation is more autonomous and less teacher-dependent than that of younger learners (Ushioda, 2011), may rely less on the affective spillover of teacher appreciation when deciding whether to communicate in unstructured settings. An alternative explanation is that the teacher appreciation dimension of FLE indexes instructor-student relational satisfaction specifically, which is simply not activated by AI interactions regardless of context, rather than representing a generalized affective resource capable of cross-contextual transfer. Distinguishing among these accounts would require studies that directly measure relational satisfaction and perceived evaluation alongside FLE dimensions.

### Social enjoyment: significant but attenuated outside the classroom

Social enjoyment contributed to mediation in both contexts, but with notably reduced strength out of class (13 to 14% vs. 23 to 25% in class). This gradient suggests that the peer interaction component of FLE is partially anchored in the social infrastructure of the classroom. AI-IDLE does generate social satisfaction through features such as discussion communities and collaborative tasks (Lee et al., 2023b; Seddik, 2025), yet this virtual social enjoyment appears to translate into face-to-face communicative willingness less efficiently than it translates into classroom WTC. One plausible explanation is that the social bonds formed through AI-mediated interaction remain ‘thin' relative to those cultivated through repeated in-person contact (Granovetter, 1973), limiting their capacity to motivate communicative risk-taking in real-world encounters. This interpretation aligns with research on the transfer gap between online and offline social capital (Ellison et al., 2007) and suggests that AI tools seeking to enhance out-of-class WTC through social pathways would benefit from incorporating features that bridge virtual and face-to-face interaction, such as AI-facilitated tandem learning partnerships that culminate in in-person meetings.

### Differential contributions of receptive and productive AI-IDLE

Productive AI-IDLE consistently showed stronger associations with all three FLE dimensions than receptive AI-IDLE, and its total indirect effects on WTC were correspondingly larger. This asymmetry is theoretically coherent: productive activities (e.g., conversational practice with AI chatbots, AI-assisted writing) require active language output that more closely approximates the demands of real communication, thereby generating a richer affective experience and a more direct transfer to communicative willingness (Soyoof et al., 2021). From the perspective of Swain's output hypothesis (Swain, 1995), productive engagement forces learners to test their interlanguage hypotheses and notice gaps in their communicative repertoire, processes that, when scaffolded by AI feedback, enhance both perceived competence and intrinsic enjoyment. This finding implies that AI tool designers should prioritize interactive, output-oriented features over passive content delivery if the goal is to maximize the affective and communicative returns of informal digital learning.

### The persistence of direct effects

In both models, significant direct effects of AI-IDLE on WTC survived after accounting for the three FLE mediators, indicating partial rather than full mediation. The direct effects were substantially larger in the out-of-class model (β = 0.18 to 0.21) than in the in-class model (β = 0.08 to 0.12). This residual direct pathway likely reflects mechanisms not captured by the FLE construct, such as gains in linguistic self-confidence (MacIntyre et al., 1998; Shirvan et al., 2019)' reductions in foreign language anxiety (Dewaele and MacIntyre, 2014; Dewaele et al., 2019), or improvements in perceived communicative competence that AI-IDLE provides independently of enjoyment. The stronger direct effects in out-of-class settings may indicate that the practical language skills acquired through AI-IDLE (e.g., vocabulary, fluency, pragmatic routines) become particularly salient when learners face the genuine communicative demands of unstructured encounters, where competence-based confidence matters more than classroom-activated positive affect. Future research incorporating anxiety and self-efficacy as additional mediators would help clarify the composition of these residual pathways.

### Theoretical contributions

This study makes three theoretical contributions. First, it introduces and empirically validates the dimensional specificity framework for FLE in technology-mediated learning. Prior work has pre-dominantly treated FLE as a unitary construct (Liu et al., 2025a,b) or, when disaggregating dimensions, has not simultaneously modeled multiple mediators across dual communicative contexts. The present findings demonstrate that collapsing FLE dimensions obscures meaningful heterogeneity in the affective pathways linking AI use to communicative behavior, and they establish a precedent for dimension-level analysis in future PP-SLA research.

Second, the study integrates the broaden-and-build theory with self-determination theory to generate dimension-specific predictions about how AI technological features map onto basic psychological needs and, in turn, onto distinct FLE dimensions. This integrated framework moves beyond the descriptive observation that “AI enhances positive emotions” to specify *which* emotions are activated by *which* features and in *which* contexts those emotions translate into communicative behavior. The result is a more explanatory and falsifiable theoretical architecture than has been available in the AI-assisted language learning literature.

Third, the dual-context design responds to repeated calls in the WTC literature for research that examines situational variability (MacIntyre et al., 1998; Denies et al., 2015). By directly comparing in-class and out-of-class WTC within the same sample and analytical framework, the study reveals that the mediating architecture is not merely weaker outside the classroom but structurally different, with teacher appreciation dropping out entirely. This context-dependent restructuring of mediation pathways adds empirical substance to the theoretical claim that WTC is a dynamic, situationally sensitive construct.

### Practical implications

The findings carry several implications for AI tool designers, educators, and curriculum developers. Given the centrality of personal enjoyment, AI learning platforms should invest in features that maximize intrinsic pleasure: adaptive difficulty algorithms, gamified progress tracking, and user-driven content selection that respects learner autonomy. These design choices are not cosmetic; the data suggest they are the primary affective lever through which AI-IDLE influences communicative behavior across all settings.

For classroom instruction, the significant mediating role of teacher appreciation indicates that AI should be positioned as a tool that amplifies, rather than replaces, teacher presence. Educators can use AI-generated analytics to deliver more targeted and timely feedback, thereby strengthening students' perception of teacher support. When teachers integrate AI into classroom tasks (e.g., AI-assisted group projects with real-time teacher moderation), the resulting synergy between technological and human scaffolding can activate all three FLE dimensions simultaneously, maximizing the affective return on AI investment.

For out-of-class learning, the data counsel a different strategy. Because teacher appreciation does not mediate WTC in informal settings, efforts to enhance out-of-class communication should focus on personal enjoyment (through personalized, output-oriented AI activities) and social enjoyment (through AI-facilitated peer communities that include mechanisms to transfer virtual rapport to real-world encounters). As a speculative direction beyond the present data, this might involve AI chatbot interactions that conclude with prompts to practice the same topic with a human partner, or AI-matched language exchange programmes that blend asynchronous digital practice with periodic face-to-face meetups.

### Limitations and future directions

Several limitations qualify the interpretation of these findings. First, the cross-sectional design precludes causal inference. Although the mediation models are informed by theory and the temporal logic of the hypothesized process (AI use precedes affective experience, which precedes communicative behavior), the data were collected at a single time point, and reverse causation or reciprocal effects cannot be ruled out. Longitudinal panel designs or experience sampling methods that capture within-person fluctuations in AI use, FLE, and WTC over time would strengthen causal claims considerably (MacIntyre and Wang, 2021). Second, all variables were assessed via self-report, raising the possibility of common method variance. Although both Harman's test and the CFA-based marker approach suggested that CMV did not substantially inflate the observed relationships, future studies could incorporate behavioral indicators of WTC (e.g., speech turn frequency, initiation rates) and objective measures of AI engagement (e.g., log data from AI platforms) to triangulate the self-report findings. Third, the sample was drawn from non-English-major undergraduates at Chinese universities, among whom 76.4% self-assessed at a beginner proficiency level. The generalizability of the dimensional specificity pattern to more advanced learners, English majors, or learners in contexts with greater exposure to natural L2 environments (e.g., ESL rather than EFL settings) remains an open question. Additionally, the pronounced beginner skew (76.4%) and the limited four-level proficiency scale may have compressed variance and constrained detection of proficiency-related moderating effects; replication with more proficiency-diverse samples and more granular assessment instruments is warranted. Cross-cultural replication, particularly in educational systems with different pedagogical traditions regarding teacher authority and classroom interaction norms, would test the robustness of the teacher appreciation asymmetry. Fourth, the AI-IDLE measure captures the frequency of receptive and productive AI-mediated activities but does not differentiate among specific AI tools (e.g., ChatGPT vs. Duolingo vs. AI pronunciation coaches) or specific technical features (e.g., real-time error correction vs. dialogue generation vs. adaptive content recommendation). A more fine-grained operationalization of AI-IDLE that maps particular technological affordances to particular FLE dimensions would advance the theoretical framework proposed here and provide more actionable guidance for tool design. Fifth, the theoretical framework did not include foreign language anxiety, self-efficacy, or perceived communicative competence as additional mediators or moderators. The significant residual direct effects observed in both models suggest that FLE does not exhaust the psychological mechanisms linking AI-IDLE to WTC. Integrating these constructs into an expanded multi-mediator model, and testing whether FLE dimensions interact with anxiety in predicting WTC, would yield a more comprehensive account of the affective architecture of AI-assisted language learning. A further limitation concerns the contextual fit between the FLE measure and the predictor. The FLE scale includes items referencing classroom settings (e.g., teacher encouragement, shared class topics), whereas AI-IDLE primarily captures out-of-class behavior. This mismatch is most salient in the out-of-class mediation model. Broaden-and-build theory posits that positive emotions accumulate durable resources that transfer across contexts over time, and personal enjoyment items (e.g., “I enjoy learning English”) are not inherently anchored to classroom settings and can reasonably reflect affect generated across learning contexts including AI-mediated informal learning. Nonetheless, future studies should adapt FLE items specifically to AI-mediated informal learning contexts.

## Conclusion

This study demonstrates that foreign language enjoyment is not a monolithic mediator but a multidimensional construct whose constituent dimensions channel the effects of AI-mediated informal digital learning onto communicative willingness through context-dependent pathways. Personal enjoyment operates as a robust cross-contextual mediator, teacher appreciation functions exclusively within structured classroom environments, and social enjoyment contributes across settings with attenuated strength outside the classroom. At the theoretical level, these findings validate the dimensional specificity hypothesis and establish a more granular framework for understanding how positive emotions mediate technology-enhanced learning and communicative behavior, extending both broaden-and-build theory and the situational variability account of WTC. At the practical level, the results indicate that personal enjoyment is the most reliable affective lever for promoting L2 WTC across learning ecologies, and that out-of-class communicative willingness is best fostered through features that support intrinsic engagement and bridge AI-facilitated interaction to real-world communicative practice.

## Data Availability

The raw data supporting the conclusions of this article will be made available by the authors, without undue reservation.

## References

[B1] Alrabai F. (2024). Modeling the relationship between classroom emotions, motivation, and learner willingness to communicate in EFL: applying a holistic approach of positive psychology in SLA research. J. Multilingual Multicul. Develop. 43, 1–19. doi: 10.1080/01434632.2022.2053138

[B2] Balouchi S. Samad A. A. (2021). The effect of perceived competence on second language communication frequency: the mediating roles of motivation, willingness to communicate, and international posture. Educ. Info. Technol. 26, 1–21. doi: 10.1007/s10639-021-10579-z

[B3] Benson P. (2011). “Language learning and teaching beyond the classroom: an introduction to the field,” in Beyond the Language Classroom, eds. P. Benson and H. Reinders, (London: Palgrave Macmillan), p. 7–16.

[B4] Botes K. Dewaele J.-M. Greiff S. (2021). The development and validation of the short form of the foreign language enjoyment scale. Modern Lang. J. 105, 858–876. doi: 10.1111/modl.12741

[B5] Brown J. S. Collins A. Duguid P. (1989). Situated cognition and the culture of learning. Educ. Res. 18, 32–42. doi: 10.3102/0013189X018001032

[B6] Cai W. (2023). ChatGPT can be a powerful tool for language learning. University Affairs. Available online at: https://www.universityaffairs.ca/articles/chatgpt-can-be-a-powerful-tool-for-language-learning/ (Accessed March 26, 2026).

[B7] Cong-Lem N. Soyoof A. Tsering D. (2025). A systematic review of the limitations and associated opportunities of ChatGPT. Int. J. Human–Computer Interact. 41, 3851–3866. doi: 10.1080/10447318.2024.2344142

[B8] Deci E. L. Ryan R. M. (1985). Intrinsic Motivation and Self-Determination in Human Behavior. New York, NY: Plenum Press. doi: 10.1007/978-1-4899-2271-7

[B9] Denies K. Yashima T. Janssen R. (2015). Classroom versus societal willingness to communicate: Investigating French as a second language in Flanders. Modern Lang. J. 99, 718–739. doi: 10.1111/modl.12276

[B10] Dewaele J.-M. (2022). “Enjoyment,” in The Routledge Handbook of Second Language Acquisition and Individual Differences, eds. S. Li, P.Hiver, andM. Papi, (Oxfordshire: Routledge). p. 190–206.

[B11] Dewaele J.-M. Chen X. Padilla A. M. Lake J. (2019). The flowering of positive psychology in foreign language teaching and acquisition research. Front. Psychol. 10, 2128. doi: 10.3389/fpsyg.2019.02128 31607981 PMC6769100

[B12] Dewaele J.-M. MacIntyre P. D. (2014). The two faces of Janus? Anxiety and enjoyment in the foreign language classroom. Stud. Second Lang. Learn. Teach. 4, 237–274. doi: 10.14746/ssllt.2014.4.2.5

[B13] Dewaele J.-M. Pavelescu M. (2019). The relationship between incommensurable emotions and willingness to communicate in English as a foreign language: a multiple case study. Innov. Lang. Learn. Teach. 5, 66–80. doi: 10.1080/17501229.2019.1675667

[B14] Dewaele J.-M. Witney J. Saito K. Dewaele L. (2018). Foreign language enjoyment and anxiety: The effect of teacher and learner variables. Lang. Teach. Res. 22, 676–697. doi: 10.1177/1362168817692161

[B15] Ellison N. B. Steinfield C. Lampe C. (2007). The benefits of Facebook “friends:” Social capital and college students' use of online social network sites. J. Computer-Mediated Communic. 12, 1143–1168. doi: 10.1111/j.1083-6101.2007.00367.x

[B16] Fornell C. Larcker D. F. (1981). Evaluating structural equation models with unobservable variables and measurement error. J. Market. Res. 18, 39–50. doi: 10.1177/002224378101800104

[B17] Fredrickson B. L. (2001). The role of positive emotions in positive psychology: the broaden-and-build theory of positive emotions. Am. Psychol. 56, 218–226. doi: 10.1037/0003-066X.56.3.218 11315248 PMC3122271

[B18] Fredrickson B. L. (2003). The value of positive emotions. Am. Sci. 91, 330–335. doi: 10.1511/2003.26.330

[B19] Granovetter M. S. (1973). The strength of weak ties. Am. J. Sociol. 78, 1360–1380. doi: 10.1086/225469

[B20] Kline R. B. (2023). Principles and practice of structural equation modeling (5th ed.). New York, NY: Guilford Press.

[B21] Kohnke L. Moorhouse B. L. Zou D. (2023). ChatGPT for language teaching and learning. RELC J. 54, 537–550. doi: 10.1177/00336882231162868

[B22] Lai C. Li Z. Gong Y. (2016). Teacher agency and professional learning in cross-cultural teaching contexts: accounts of Chinese teachers from international schools in Hong Kong. Teach. Teach. Educ. 54, 12–21. doi: 10.1016/j.tate.2015.11.007

[B23] Lee J. S. (2022). The role of informal digital learning of English in L2 willingness to communicate. J. Multilingual Multicul. Devel. 43, 1–15.

[B24] Lee J. S. Hsieh J. C. (2019). Informal digital learning of English: a systematic review. Comp. Assist. Lang. Learn. 35, 1–28.

[B25] Lee J. S. Shin S.-K. Noh S. (2023a). AI chatbot-assisted language learning: effects on learner enjoyment, anxiety, and willingness to communicate. Educ. Technol. Soc. 26, 98–113.

[B26] Lee J. S. Xie Q. Lee K. (2023b). Informal digital learning of English and L2 willingness to communicate: roles of emotions and gender. British J. Educ. Technol. 54, 291−313.

[B27] Lewis M. Haviland-Jones J. M. Barrett L. F. (eds.). (2016). Handbook of Emotions, 4th Edn. The Guilford Press.

[B28] Li C. Jiang G. Dewaele J.-M. (2018). Understanding Chinese high school students' foreign language enjoyment: validation of the Chinese version of the foreign language enjoyment scale. System 76, 183–196. doi: 10.1016/j.system.2018.06.004

[B29] Liu G. L. Darvin R. Ma C. (2025a). Exploring AI-mediated informal digital learning of English (AI-IDLE): a mixed-method investigation of Chinese EFL learners' AI adoption and experiences. Computer Assisted Lang. Learn. 38, 1632–1660. doi: 10.1080/09588221.2024.2310288

[B30] Liu G. L. Zou M. M. Soyoof A. Chiu M. M. (2025b). Untangling the relationship between AI-mediated informal digital learning of English (AI-IDLE), foreign language enjoyment and the ideal L2 self: evidence from Chinese university EFL students. Eur.J. Educ. 60:e12846. doi: 10.1111/ejed.12846

[B31] MacIntyre P. D. Clément R. Dörnyei Z. Noels K. A. (1998). Conceptualizing willingness to communicate in a L2: a situational model of L2 confidence and affiliation. Modern Lang. J. 82, 545–562. doi: 10.1111/j.1540-4781.1998.tb05543.x

[B32] MacIntyre P. D. Gregersen T. Mercer S. (2019). Setting an agenda for positive psychology in SLA: theory, practice, and research. Modern Lang. J. 103, 262–274. doi: 10.1111/modl.12544

[B33] MacIntyre P. D. Wang Z. (2021). Willingness to communicate in the L2 about meaningful photos: application of the pyramid model of WTC. Lang. Teach. Res. 25, 878–898. doi: 10.1177/13621688211004645

[B34] Mehdi Y. (2023). Reinventing search with a new AI-powered Microsoft Bing and edge, your copilot for the web. Official Microsoft Blog. Available online at: https://news.microsoft.com/the-new-Bing/ (Accessed March 26, 2026).

[B35] Muthén L. K. Muthén B. O. (2017). Mplus user's guide: statistical analysis with latent variables (8th ed.). Los Angeles, CA: Muthén and Muthén.

[B36] Nematizadeh S. (2021). Willingness to communicate and second language speech fluency: An idiodynamic investigation of attractor states. J. Psychol Lang. Learn. 3, 26–49. doi: 10.52598/jpll/3/1/2

[B37] Noels K. A. Pelletier L. G. Clément R. Vallerand R. J. (2000). Why are you learning a second language? Motivational orientations and self-determination theory. Lang. Learn. 50, 57–85. doi: 10.1111/0023-8333.00111

[B38] Podsakoff P. M. MacKenzie S. B. Lee J.-Y. Podsakoff N. P. (2003). Common method biases in behavioral research: a critical review of the literature and recommended remedies. J. Appl. Psychol. 88, 879–903. doi: 10.1037/0021-9010.88.5.879 14516251

[B39] Preacher K. J. Hayes A. F. (2008). Asymptotic and resampling strategies for assessing and comparing indirect effects in multiple mediator models. Behav. Res. Methods. 40, 879–891. doi: 10.3758/BRM.40.3.879 18697684

[B40] Rezai A. Soyoof A. Reynolds B. L. (2024). Disclosing the correlation between using ChatGPT and well-being in EFL learners: considering the mediating role of emotion regulation. Eur. J. Educ. 59:e12752. doi: 10.1111/ejed.12752

[B41] Rudolph J. Tan S. Tan S. (2023). ChatGPT: bullshit spewer or the end of traditional assessments in higher education? J. Appl. Learn. Teach. 6, 342–363. doi: 10.37074/jalt.2023.6.1.9

[B42] Ryan R. M. Deci E. L. (2000). Self-determination theory and the facilitation of intrinsic motivation, social development, and well-being. Am. Psychol. 55, 68–78. doi: 10.1037/0003-066X.55.1.68 11392867

[B43] Seddik M. E. (2025). The impact of AI-powered language learning tools on second language acquisition: a mixed-methods study. Int.J. Linguistics, Literature Transl. 8, 269–278. doi: 10.32996/ijllt.2025.8.3.30

[B44] Shirvan M. E. Khajavy G. H. MacIntyre P. D. Taherian T. (2019). A meta-analysis of L2 willingness to communicate and its three high-evidence correlates. J. Psycholinguis. Res. 48, 1241–1267. doi: 10.1007/s10936-019-09656-9 31342240

[B45] Soyoof A. Reynolds B. L. Vazquez-Calvo B. McLay K. (2021). Informal digital learning of English (IDLE): a scoping review of what has been done and a look towards what is to come. Computer Assisted Lang. Learn. 36, 608–640. doi: 10.1080/09588221.2021.1936562

[B46] Swain M. (1995). “Three functions of output in second language learning,” in Principles and Practice in Applied Linguistics: Studies in Honor of H. G. Widdowson, eds. G. Cook and B. Seidlhofer (Oxford: Oxford University Press), p. 125–144.

[B47] Tai T.-Y. Chen H. H. (2023). The impact of Google Assistant on adolescent EFL learners' willingness to communicate. Interact. Learn. Environ. 31, 1485–1502. doi: 10.1080/10494820.2020.1841801

[B48] Ushioda E. (2011). “Motivating learners to speak as themselves,” in Identity, Motivation and Autonomy in Language Learning, eds. G. Murray, X. Gao, and T. Lamb, (Bristol: Multilingual Matters), p. 11–25.

[B49] Wang H. Peng A. Patterson L. (2021). The roles of class social climate, language mindset, and emotions in predicting willingness to communicate in a foreign language. System 99:102529. doi: 10.1016/j.system.2021.102529

[B50] Wang X. Gao Y. Reynolds B. L. (2026). L2 self-guides, achievement emotions, and engagement in AI-mediated informal digital learning of English: insights from structural equation modeling and psychological network analysis. The Asia-Pacific Education Researcher. New York, NY: Advance Online Publication. doi: 10.1007/s40299-025-01073-y

[B51] Wang X. Gao Y. Reynolds B. L. Wang Q. (2025). Exploring Chinese EFL learners' beliefs about AI-mediated informal digital learning of English (IDLE): insights from Q methodology. Porta Linguarum 13, 131–146. doi: 10.30827/portalin.viXIII.31925

[B52] Wu X. Y. (2024). Artificial intelligence in L2 learning: A meta-analysis of contextual, instructional, and social-emotional moderators. System 126:103498. doi: 10.1016/j.system.2024.103498

[B53] Yan D. (2023). Impact of ChatGPT on learners in a L2 writing practicum: an exploratory investigation. Educ. Info. Technol. 28, 13943–13967. doi: 10.1007/s10639-023-11742-4

[B54] Zhang C. Meng Y. Ma X. (2024). Artificial intelligence in EFL speaking: impact on enjoyment, anxiety, and willingness to communicate. System 121:103259. doi: 10.1016/j.system.2024.103259

[B55] Zhang Y. Liu G. (2024). Revisiting informal digital learning of English (IDLE): a structural equation modeling approach in a university EFL context. Computer Assisted Lang. Learn. 37, 1904–1936. doi: 10.1080/09588221.2022.2134424

