# Supplementary material for: Dimensional specificity of foreign language enjoyment in mediating AI-assisted informal digital learning and L2 willingness to communicate: evidence from Chinese University learners
Source: Front Psychol. 2026 May 4;17:1817760. doi: 10.3389/fpsyg.2026.1817760 (PMC13180624; doi:10.3389/fpsyg.2026.1817760)
Supplement: Supplementary file 1 [file Table_1.pdf]

Supplementary Table S1

Descriptive Statistics for AI-IDLE Variables Before and After Exclusion of Non-Users

Variable	Group	n	*M*	*SD*	Skewness
Receptive AI-IDLE	Full sample (N = 1,562)	1,562	3.04	1.07	−0.08
	AI users – retained (n = 1,362)	1,362	3.28	0.91	−0.31
	Non-users – excluded (n = 200)	200	1.38	0.44	0.62
Productive AI-IDLE	Full sample (N = 1,562)	1,562	3.19	1.16	−0.05
	AI users – retained (n = 1,362)	1,362	3.47	0.96	−0.29
	Non-users – excluded (n = 200)	200	1.32	0.40	0.68

Note. AI-IDLE = AI-mediated informal digital learning of English. The full sample comprised 1,562 participants prior to exclusion. Non-users (n = 200) were defined as respondents who reported never having used generative AI tools for English learning;
